# Marital Adjustment in Patients with Cancer: Association with Psychological Distress, Quality of Life, and Sleep Problems

**DOI:** 10.3390/ijerph18137089

**Published:** 2021-07-02

**Authors:** Carmen Maria Ruiz-Marin, Rocio Molina-Barea, Mahmoud Slim, Elena P. Calandre

**Affiliations:** 1 Servicio de Cirugía, Complejo Hospitalario de Jaen, 23007 Jaen, Spain; carmenmariaruizmarin@hotmail.com (C.M.R.-M.); barea1984@gmail.com (R.M.-B.); 2 Division of Neurology, The Hospital for Sick Children, Toronto, ON M5G 0A4, Canada; mahmoud.slim@gmail.com; 3 Instituto de Neurociencias, Universidad de Granada, 18100 Granada, Spain

**Keywords:** marital adjustment, cancer, psychosocial, depression, anxiety, quality of life

## Abstract

Marital adjustment plays a key role in the physical and psychosocial wellbeing. We conducted a cross-sectional study to evaluate marital adjustment and its association with psychological distress, suicidal ideation, sleep problems, and quality of life in patients with cancer. We collected demographic and clinical information using a structured survey. We assessed marital adjustment, quality of life, psychological distress profile, and sleep problems of participants using validated instruments: the Locke and Wallace Marital Adjustment Test (LWMAT), the Short-Form Health Survey-12, the Beck’s Depression Inventory (BDI), the Beck Anxiety Inventory (BAI), and the Insomnia Severity Index (ISI). Suicidal ideation was assessed with item nine of the BDI. Of the 130 patients (52.3% females, mean age 57.9 ± 12.4 years) enrolled, 20 (15%) were classified as experiencing poor marital adjustment. Moderate to severe depression, anxiety, and insomnia were found in 25.4%, 34.6%, and 24.7% of participants, respectively. Positive suicidal ideation was documented in 13.8% of participants. We found a significant association between poor marital adjustment and depression, anxiety, suicidal ideation, and poor sleep. Our study confirms the relevance of marital adjustment in relation to the psychological wellbeing of patients with cancer. Depression, anxiety, and poor sleep were found to be significantly associated with poor marital adjustment.

## 1. Introduction

Marital adjustment is defined as a process whose outcome is determined by the extent of troublesome marital differences, interspousal tensions, personal anxiety, marital satisfaction, and dyadic cohesion [1]. Marital adjustment plays a key role in the physical and psychosocial wellbeing of partners, with higher levels being linked to better health outcomes [2,3]. On the other hand, poor adjustment does not only lead to poor health outcomes but also to increased odds of mortality [4]. Poor marital adjustment has been linked to increased morbidity and mortality across various chronic disease conditions, including cancer [5,6].

The incidence of cancer is rapidly growing worldwide, leading to an estimated 9.6 million deaths in 2018 [7]. While advances in cancer detection and treatment have led to significant improvement in survival rates, associated psychiatric illnesses such as depression and anxiety remain commonly overlooked despite their influence on the patients’ quality of life and prognosis [8,9]. It has been demonstrated that psychological distress is not confined to the individual diagnosed with cancer but extends to affect all family members [10]. In particular, findings from the meta-analysis conducted by Hodges et al. confirmed the presence of close association and mutuality in psychological response between cancer patients and their partners [11]. Thus, it has been suggested that the patient–partner relationship should be also addressed as part of cancer support services [12].

The relationship between marital status and cancer has been evaluated in several studies [13,14,15,16,17,18], with findings strongly suggesting that being married or in a long-term partnership is a prognostic factor of better survival, both in relation to all-cause mortality and cancer-specific mortality death. This has been largely attributed to the psychosocial support provided by the spouse, which contributes to a reduction in the psychological distress associated with cancer diagnoses [13,14,15,16,17,18]. However, all of these studies were done by extracting data from different databases in which marital status was indicated but no data were available concerning the quality of the marriage. Not every marriage is a happy one and the degree of marital adjustment between partners has been scarcely considered in the field of cancer research. Moreover, for the same methodological reason, no study evaluated subjects living with a partner but not married.

In the context of breast cancer, several studies have demonstrated that high levels of marital adjustment are associated with better functioning and quality of life [19]. On the contrary, poor adjustment has been shown not only to be associated with worse psychological outcomes but also with poorer health and a steeper decline in physical activity [20]. In another study that included other types of cancer, greater functional impairment was associated with higher levels of patient psychological distress, which in turn correlated significantly with lower marital satisfaction as rated by the spouse [21]. However, the association of marital satisfaction, as assessed by the patient, with the patient’s psychological distress and quality of life outside the context of breast cancer remains poorly investigated.

The prevalence of psychological distress among cancer patients in Spain has been scarcely investigated and, to our knowledge, no study has addressed the impact of marital adjustment nor satisfaction in relation to this issue. Therefore, in our current study, we sought to study the prevalence of psychological distress in a sample of Spanish patients diagnosed with cancer and to evaluate the extent of association between marital adjustment with the patients’ psychological distress, suicidal ideation, sleep problems, and quality of life.

## 2. Materials and Methods

### 2.1. Study Design

We conducted a cross-sectional study at the Oncology Division of the Jaen University Hospital in Spain between February 2018 to February 2019. Written informed consent was obtained from all study participants. The study protocol was approved by the Human Research Ethics Committee at the University Hospital of Jaen (Reference number: EAM1/1819-N-20). 

### 2.2. Subjects and Procedures

The study sample comprised of adult patients with documented cancer diagnoses. Patients attending the outpatient clinics at the Jaen University Hospital were screened for eligibility. Inclusion criteria included: (1) males or females aged >18 years of age, with (2) a documented diagnosis of cancer of any type and who are clinically stable, and (3) living with a spouse or stable partner. Patients were excluded if they did not consent to participation or if they were unable to understand or complete the study questionnaires. Patients were given a form with facts concerning the purpose of the study and the potential benefits of gathering information about the relationship between the marital satisfaction and the psychological wellbeing of patients. Patients were informed that their participation would be voluntary, and that if they preferred not to participate this decision would not influence the medical care they received, and that the collected data would be anonymous. The questionnaires were given out by one of the study investigators in printed form, to be completed and returned on the same day.

### 2.3. Outcome Measures

#### 2.3.1. Marital Adjustment

Marital adjustment was evaluated using the Locke and Wallace Marital Adjustment Test (LWMAT) [22]. Consisting of 15 items, the LWMAT measures general marital quality or satisfaction, as well as agreement or disagreement on common issues that cause conflict for couples. The total score ranges from 2 to 158, with higher scores indicating better marital satisfaction. The Cronbach’s alpha coefficient of the test is 0.91 [22]. A cut-off point of 94 is indicative of a good marital adjustment, as shown in the Spanish-validated version of the test [23].

#### 2.3.2. Quality of Life

The quality of life was assessed using the Short-Form Health Survey-12 (SF-12). The SF-12 consists of 12 items and is a subset of the 36-item Short Form Health Survey [24]. Two component scores are reported based on the patient’s responses: the physical component score (PCS) and the mental component score (MCS). The total score for each component ranges between 0 and 100, with higher scores indicating better quality of life. Within the Spanish population, the Cronbach’s alpha has been shown to be 0.85 for the PCS and 0.78 for the MCS [25].

#### 2.3.3. Depressive Symptomatology

Depressive symptomatology was assessed using the Beck’s Depression Inventory, second edition (BDI-II) [26]. BDI-II is a self-reported questionnaire consisting of 21 items. Total scores range between 0 and 63, with higher scores indicating more severe symptomatology. Range values of 0–13 are considered minimal depression; 14–19 mild depression; 20–28 moderate depression; and 29–63 severe depression [27]. The Cronbach’s alpha has been estimated to be 0.92 for outpatients and 0.93 for college students [27].

#### 2.3.4. Suicidal Ideation

Suicidal ideation was assessed using the ninth item of the Beck’s Depression Inventory, second edition [26]. Item nine of the BDI-II has four response levels: 0 (I don’t have any thoughts of harming myself); 1 (I have thoughts of harming myself but would not carry them out); 2 (I would like to kill myself); and 3 (I would kill myself if I had the chance). Patients who score ≥1 are considered to have a positive suicidal ideation. The use of this item of the BDI to assess suicidal ideation is a common tool that is frequently employed [28,29].

#### 2.3.5. Anxiety

The severity of anxiety was assessed using the self-administered Beck Anxiety Inventory (BAI), which is a 21-item survey [30]. The total score ranges between 0 and 63, with higher scores indicating more severe anxiety. Range values of 0–9 are considered normal or no anxiety; 10–18, mild to moderate anxiety; 19–29, moderate to severe anxiety; and 30–63, severe anxiety [31]. Its Cronbach’s alpha within the general Spanish population has been estimated to be 0.93 [32].

#### 2.3.6. Sleep Problems

We used the Insomnia Severity Index (ISI) to study sleep problems. It is a brief seven-item instrument which is used to evaluate the patient’s perceptions of insomnia severity and its impact on daily life function. It has shown a Cronbach’s alpha of 0.90 in the general population and 0.91 in patients with insomnia [33]. The total score ranges between 0 and 28, in which higher scores indicate a greater degree of insomnia. Range values from 0–7 indicate no clinically significant insomnia; 8–14, subthreshold insomnia; 15–21, clinical insomnia of moderate severity; and 22–28, severe clinical insomnia [34].

### 2.4. Statistical Analysis

Demographic and clinical characteristics were examined using descriptive statistics. Continuous variables were presented as mean ± SD or median and interquartile ranges (IQR), as appropriate. We examined the normal distribution of the continuous variables using the Shapiro–Wilk test. Qualitative variables were presented using frequency distributions and percentages.

For the continuous variables, group comparisons were conducted using the Student’s *t*-test or Wilcoxon–Mann–Whitney test, as appropriate. Differences in qualitative outcome variables were assessed using χ2 or the Fisher exact test, as appropriate. We used linear regression models to identify the demographic and clinical variables that correlated with marital adjustment (assessed using LWMAT scores). We used multiple linear regression models to control for age, sex, and the presence of comorbid medical conditions. We used the bootstrapping residual resampling procedure to calculate approximate 95% confidence intervals (CIs) for the regression coefficients. All statistical tests were two-sided, with *p* < 0.05 considered to be statistically significant. Statistical analysis was conducted using R statistical and computing software, Version 3.4.1 (http://www.r-project.org, accessed on 1 May 2021) and GraphPad Prism, Version 7 (GraphPad Software, San Diego, CA, USA).

## 3. Results

### 3.1. Study Sample

A total of 130 patients completed the study questionnaires. Their sociodemographic and clinical characteristics are summarized in Table 1. With respect to type of cancer, breast cancer was the most common (*n* = 33, 25.4%), followed by colon and lung cancer (24.6% and 7.7%, respectively). Most of the patients were recently diagnosed (*n* = 85, 65.4%), i.e., within the last year.

In general, the study sample displayed a favorable marital adjustment profile. Only 20 patients (15%) were classified as experiencing poor marital adjustment. Moderate to severe depression, anxiety, and insomnia were found in 25.4%, 34.6%, and 24.7% of the total sample, respectively. Positive suicidal ideation was documented in 18 patients (13.8%).

### 3.2. Differences between Patients with Good versus Poor Marital Adjustment

Patients with poor and those with good marital adjustment had comparable sociodemographic characteristics (Table 2). At the level of clinical characteristics, although non-statistically significant, patients with poor marital adjustment were more likely to have received their diagnosis since ≥1 year since enrollment compared to patients with good marital adjustment (50% versus 31.8%, *p* = 0.11). Breast, colon, and lung cancer were more common among patients with poor marital adjustment (*p* = 0.16). On the other hand, patients with poor adjustment were more likely to have undergone surgical intervention (90% versus 10%, *p* = 0.02) and to be diagnosed with other comorbid conditions (65% versus 40%, *p* = 0.04).

While patients with good marital adjustment showed significantly better SF-12 MCS scores compared to those with poor adjustment (43.7 ± 11.8 versus 33.3 ± 7.6, *p* < 0.001), no significant differences were found at the level of PCS scores.

Patients with poor marital adjustment had significantly worse depressive symptomatology, with a mean BDI-II score of 19.9 ± 12.2 compared with 12.3 ± 8.2 in patients with good marital adjustment (*p* = 0.003). In addition, more than half of the patients with poor marital adjustment had moderate to severe depression, compared with only 19% of patients with good marital adjustment. Similar findings were observed at the level of anxiety whereby patients with poor marital adjustment had significantly higher BAI scores as compared to patients with good marital adjustment (20.8 ± 14.2 versus 14 ± 11.3, *p* = 0.04) (Table 2). Suicidal ideation was remarkably more common among patients with poor marital adjustment (30% versus 11%, *p* = 0.03). In addition, patients with poor marital adjustment had worse sleep problems where they had significantly higher ISI scores compared to those with good marital adjustment (14.4 ± 7 versus 9.6 ± 6.5, *p* = 0.008).

### 3.3. The Impact of Demographic, Clinical, and Psychosocial Characteristics on Marital Adjustment

As shown in Table 3, a history of surgical intervention, depressive symptomatology, suicidal ideation, anxiety, and poor sleep were all associated with significantly negative impacts on marital adjustment. Higher SF-12 MCS scores were associated with significant positive impacts on favorable marital adjustment. When controlling for age, sex, and comorbid medical conditions, the history of surgical intervention and MCS outcomes were no longer associated with marital adjustment. Results remained statistically significant for the remaining psychosocial outcomes.

## 4. Discussion

Although substantial attention has been paid to the relationship between marital status and psychological functioning in patients with cancer [35], to our knowledge, this is the first study that evaluates the different symptoms of psychological distress, such as anxiety, depression, sleep problems, and suicidal ideation as well as health-related quality of life in a sample of patients with cancer in relation to their degree of marital adjustment. In our current study, more than one third of the patients reported moderate so severe depression, anxiety, and/or sleep disturbances. While a low level of marital adjustment was relatively uncommon in our sample, when present it correlated significantly with poor quality of life, clinical, and psychosocial characteristics.

Cancer is, in most cases, a life-threatening disease, and as such it poses an important challenge for the subjects’ wellbeing. Quality of life is significantly impacted, both by the disease and by treatment-related adverse effects [36]. It is, thus, understandable that symptoms of psychosocial distress, such as anxiety, depression, and sleep problems, are frequently seen in these patients. It has been estimated that up to 20% of cancer patients show depressive symptoms and up to 10% show anxiety [37], with at least a third of them reporting sleep disturbances [38]. In our sample, 43.1% of subjects suffered from moderate to severe depression, 34.6% of subjects suffered from moderate to severe anxiety, and 24.6% of subjects suffered from moderate to severe insomnia. These percentages, however, were clearly different in patients with good and poor marital adjustment, with the latter group showing the highest proportion of subjects with moderate to severe depression (55.0%), moderate to severe anxiety (45.0%), and moderate to severe insomnia (55.0%).

Although the prevalence of psychological distress varies depending on the characteristics of the studied sample, suicidal ideation in patients with cancer has been shown to be fairly frequent [39]. Risk factors include depression, anxiety, older age, and unmarried status, as well disease and treatment-related factors [39]. In our study the prevalence in the whole sample was 18 (13.9%), which is in the lower range of the prevalence found in other studies [39], probably because the patients in our sample were of middle age and were in a clinically stable condition. However, again, the proportion of patients with suicidal ideation noticeably differed between patients with good (10.9%) and poor marital adjustment (30.0%).

Our study has demonstrated the presence of a significant association between marital adjustment and psychological distress, even after controlling for potential confounders. For instance, health-related quality of life decreases with age and is lower in patients than in healthy subjects; likewise, both anxiety and depression have been shown to be more frequent in women than in men. For this reason, we adjusted the data in relation to age, sex, and associated comorbidities. After this adjustment, the association of poor marital relationship with depression, anxiety, sleep problems, suicidal ideation, and mental health persisted; these data suggest the presence of a significant association. In this respect, Weihs et al. found, in patients with breast cancer, that a poor marital relationship was related with higher emotional distress [40].

To improve feelings of anxiety and depression and to decrease suicidal thoughts, it is usually assumed that social relationships and family support play a critical role [41,42]. In this context, marital adjustment is a relevant factor since it is unlikely that, in a distressed marital relationship, the patient’s spouse will provide emotional nor physical support to her/his partner. It has been well-established that depressed individuals with poor marital adjustment recover less quickly from their depressive episode and are more likely to experience major depression relapse [43].

As mentioned earlier in the introduction, good marital adjustment has been associated with better physical and mental health outcomes and even with lower odds for mortality. Of interest, in patients with cancer it has been shown that married patients were less likely to develop metastases and were less likely to die as a result of their disease as compared to unmarried patients [13]. Although this was attributed to improved support, the aforementioned study did not investigate the degree of marital satisfaction. Yang and Schuler, in a five-year longitudinal study, found that cancer patients experiencing marital distress, as compared with those not distressed, showed lower improvement in cancer-specific stress and had deteriorating health behaviors and impaired functioning [20]. The favorable health outcomes among cancer patients with better marital adjustment can be attributed to the better access to care, earlier diagnoses, and better adherence to therapies due to possible encouragement by spouses [13]. Access to medical care and healthcare utilization have been shown to vary based on marital status and are likely to be influenced by the level of martial adjustment. In a recently published study, married individuals had higher odds of seeking medical care in outpatient clinics compared with unmarried individuals [44]. In the context of cancer, this can have a drastic impact on disease outcomes by promoting earlier diagnosis and subsequently favorable health outcomes. Furthermore, two previous studies have demonstrated that better marital adjustment is linked to better compliance with therapy and improved disease control among patients with hypertension or diabetes [45,46]. Adherence to therapy in various types of cancer has been shown to be an important predictor of survival and health outcomes [47,48].

In women with breast cancer, marital satisfaction was found to be positively associated with favorable psychological change and health-related quality of life [49,50]. On the other hand, it was negatively correlated with depression [51] and global psychological distress [40]. Thus, although the data are still scarce and mostly related only to breast cancer, it seems that in patients with cancer a good marital relationship is a relevant factor that contributes to better health outcomes. Our results are in accordance with these findings, as anxiety, depression, and suicidal ideation were significantly higher in subjects with poor marital adjustment. In the general population, marital discord was found to be positively correlated with suicidal ideation and attempts [52]; our results found a significantly higher prevalence of suicidal ideation among patients reporting poor marital adjustment. Physical and mental component scores of the SF-12, however, did not show significant differences between good and marital adjustment groups after adjusting for age and sex; this is easily understandable given that the quality of life tends to decrease with advancing age.

Although substantial attention has been paid to the relationship between marital status and psychological functioning in patients with cancer [35], to our knowledge, this is the first study that evaluates different symptoms of psychological distress, such as anxiety, depression, and suicidal ideation as well as health-related quality of life in cancer patients in relation to their degree of marital adjustment.

One of the limitations of our study was the absence of sample-size calculations. This is primarily attributed to the pilot nature of our study. Although there was a partial overlap between our study objectives and those in the study conducted by Yang and Schuler [20], there were substantial differences in the study populations. In the latter, only female patients with breast cancer were included, whereas in our current study we included patients of both sexes with cancers of any origin. Therefore, it was not possible to conduct sample-size calculations and we decided to recruit a consecutive sample of adult patients with documented cancer diagnoses. Although our study sample size was not small, the fact that most of the couples had a good marital adjustment hindered the probability of finding differences between groups, a problem that would have been easily overcome with a larger sample size. Furthermore, our study was conducted at a single center which limits the generalizability of our study findings. Thus, the conclusion of our study should be interpreted with caution.

Another limitation of our study was the absence of a third group of patients without partners. This would have been useful to assess the impact of marital status along with the adjustment status on the health status and quality of life of patients with cancer. Nevertheless, it remains of interest to investigate how these results may vary when marital adjustment is accounted for in the analysis. In addition, it must be also taken into account that we did not gather any data of patients that refused to participate in the study; perhaps it could have been useful to collect some data such age, sex, and type of neoplasm in order to evaluate if there were any differences between patients willing to participate compared with those who refused. Additionally, as the study design was cross-sectional, we cannot establish causality; thus it is impossible to ascertain if poor marital adjustment was the determinant of psychological distress or if, on the contrary, the psychological distress influenced the degree of marital adjustment. Finally, it must be taken in account that, although all patients were clinically stable, we did not gather data related to tumor stage or the type of chemotherapy received.

## 5. Conclusions

Notwithstanding the above-mentioned limitations, the results of our study show that, as expected, a poor marital relationship was associated with higher depression and anxiety scores, a higher frequency of positive suicidal ideation, higher scores of sleep disturbance, and lower mental health scores, without differences in relation to physical heath scores. After controlling for age, sex, and comorbid medical conditions, depression, anxiety, mental health, and poor sleep were shown to be associated with a negative impact on marital adjustment. These findings highlight the relevance of marital adjustment in relation to patients’ wellbeing and encourage the need for additional studies in this area.

## Figures and Tables

**Table 1:** Sociodemographic, clinical, and psychosocial characteristics.

	Total Sample *n* = 130
Sociodemographic Characteristics	
Age in years, mean ± SD	57.9 ± 12.4
Female sex, *n* (%)	68 (52.3)
Educational status, *n* (%)	
No school	16 (12.3)
Primary school	49 (37.7)
secondary school	36 (27.7)
University	29 (22.3)
Employment status, *n* (%)	
Works only at home	15 (11.3)
Works outside home	20 (15.4)
Unemployed	8 (6.2)
Retired	31 (23.8)
Temporary sick leave	26 (20.9)
Permanent sick leave	22 (16.9)
Other	8 (6.2)
Clinical Characteristics	
Time since diagnosis, *n* (%)	
<1 year	85 (65.4)
≥1 year	45 (34.6)
Tumor type, *n* (%)	
Breast	33 (25.4)
Colon	31 (23.9)
Other gastrointestinal tumors *	29 (22.3)
Lung	10 (7.7)
Other	27 (20.8)
Surgical intervention, *n* (%)	88 (67.7)
Comorbid conditions, *n* (%)	57 (43.8)
Quality of life and Psychosocial Outcomes	
LWMAT, mean ± SD	117.1 ± 23.9
SF-12 PCS, mean ± SD	39.3 ± 9.6
SF-12 MCS, mean ± SD	42.1 ± 11.8
BDI-II, mean ± SD	13.4 ± 9.3
Severity of depression, *n* (%)	
Minimal	74 (56.9)
Mild	23 (17.7)
Moderate	25 (19.2)
Severe	8 (6.2)
Suicidal ideation, *n* (%)	18 (13.9)
BAI, mean ± SD	15 ± 11.9
Severity of anxiety, *n* (%)	
Normal (0–9)	49 (37.7)
Mild–moderate (10–18)	36 (27.7)
Moderate–severe (19–29)	28 (21.5)
Severe (30–63)	17 (13.1)
ISI, mean ± SD	10.3 ± 6.8
Severity of insomnia, *n* (%)	
No clinically significant insomnia (0–7)	53 (40.8)
Subthreshold insomnia (8–14)	45 (34.6)
Clinical insomnia of moderate severity (15–21)	24 (18.5)
Severe clinical insomnia (22–28)	8 (6.2)

SD: standard deviation; LWMAT: Locke and Wallace Marital Adjustment Test; SF-12 PCS: Short-Form Health Survey Physical Component Score; SF-12 MCS: Short-Form Health Survey Mental Component Score; BDI-II: Beck’s Depression Inventory, second edition; BAI: Beck’s Anxiety Inventory; ISI: Insomnia Severity Index. * Other GI tumors included stomach, liver, rectal, nasopharyngeal, esophageal, laryngeal, and pancreatic cancers.

**Table 2:** Comparison of sociodemographic, clinical, and psychosocial characteristics between patients with good and poor marital adjustment.

	Good Marital Adjustment *n* = 110	Poor Marital Adjustment *n* = 20	*p*-Value
Sociodemographic Characteristics			
Age in years, median (IQR)	58.5 (50–67)	57 (52–65.5)	0.55
Female sex, *n* (%)	58 (52.7)	10 (50)	0.82
Educational status, *n* (%)			0.33
No school	11 (10)	5 (25)	
Primary school	42 (38.2)	7 (35)	
secondary school	32 (29.1)	4 (20)	
University	25 (22.7)	4 (20)	
Employment status, *n* (%)			0.76
Works only at home	12 (10.9)	3 (15)	
Works outside home	18 (16.4)	2 (10)	
Unemployed	7 (6.4)	1 (5)	
Retired	25 (22.7)	6 (30)	
Temporary sick leave	24 (21.8)	2 (10)	
Permanent sick leave	18 (16.4)	4 (20)	
Other	6 (5.5)	2 (10)	
Clinical Characteristics			
Time since diagnosis, *n* (%)			0.11
<1 year	75 (68.2)	10 (50)	
≥1 year	35 (31.8)	10 (50)	
Tumor type, *n* (%)			0.16
Breast	30 (27.8)	3 (15)	
Colon	27 (24.5)	4 (20)	
Other gastrointestinal tumors	24 (21.8)	5 (25)	
Lung	10 (9.1)	0	
Other	19 (17.3)	8 (40)	
Surgical intervention, *n* (%)	70 (63.6)	18 (90)	0.02
Comorbid conditions, *n* (%)	44 (40)	13 (65)	0.04
Quality of life and Psychosocial Outcomes			
SF-12 PCS, mean ± SD	39.9 ± 9.7	36.2 ± 8.9	0.11
SF-12 MCS, mean ± SD	43.7 ± 11.8	33.3 ± 7.6	<0.001 *
BDI-II, mean ± SD	12.3 ± 8.2	19.9 ± 12.2	0.003 *
Severity of depression, *n* (%)			0.002
Minimal (0–13)	68 (61.8)	6 (30)	
Mild (14–19)	21 (19.1)	2 (10)	
Moderate (20–28)	16 (14.6)	9 (45)	
Severe (29–63)	5 (4.5)	3 (15)	
Suicidal ideation, *n* (%)	12 (10.9)	6 (30)	0.03
BAI, mean ± SD	14 ± 11.3	20.8 ± 14.2	0.04 *
Severity of anxiety, *n* (%)			0.008
Normal (0–9)	45 (40.9)	4 (20)	
Mild–moderate (10–18)	29 (26.4)	7 (35)	
Moderate–severe (19–29)	26 (23.6)	2 (10)	
Severe (30–63)	10 (9.1)	7 (35)	
ISI, mean ± SD	9.6 ± 6.5	14.4 ± 7	0.008 *
Severity of insomnia, *n* (%)			0.001
No clinically significant insomnia (0–7)	48 (43.6)	5 (25)	
Subthreshold insomnia (8–14)	42 (38.2)	3 (15)	
Clinical insomnia of moderate severity (15–21)	15 (13.6)	9 (45)	
Severe clinical insomnia (22–28)	5 (4.6)	3 (15)	

*: Data were not normally distributed; comparisons were assessed using the Wilcoxon–Mann–Whitney test. IQR: interquartile range; SD: standard deviation; SF-12 PCS: Short-Form Health Survey Physical Component Score; SF-12 MCS: Short-Form Health Survey Mental Component Score; BDI-II: Beck’s Depression Inventory, second edition; BAI: Beck Anxiety Inventory; ISI: Insomnia Severity Index.

**Table 3:** The impact of clinical and psychological characteristics on marital adjustment (LWMAT score).

	Univariate Estimates			Multivariable Estimates ^#^		
	B (95% CI)	F-Statistic (*p*-Value *)	Adjusted R2	B (95% CI)	F-statistic (*p*-Value *)	Adjusted R2
Surgical Intervention	−8.77 (−17.1, −0.89)	5.56 (0.019)	0.034	−6.6 (−15.2, 1.4)	2.72 (0.03)	0.051
SF-12 MCS	0.34 (0.013, 0.66)	5.52 (0.02)	0.033	0.31 (−0.016, 0.64)	3.09 (0.02)	0.061
SF-12 PCS	0.13 (−0.28, 0.56)	1.24 (0.26)	0.019	0.08 (−0.33, 0.52)	2.09 (0.09)	0.032
BDI-II	−0.63 (−1.04, −0.21)	7.65 (0.006)	0.049	−0.60 (−1.01, −0.19)	3.48 (0.01)	0.071
Positive suicidal ideation	−17.5 (−29.1, −5.5)	9.79 (0.002)	0.063	−17.7 (−29.2, −5.9)	4.21 (0.003)	0.091
BAI	−0.45 (−0.76, −0.12)	8.07 (0.005)	0.052	−0.35 (−0.69, −0.01)	3.11 (0.02)	0.061
ISI	−1.02 (−1.6, −0.49)	10.45 (0.001)	0.068	−0.88 (−1.5, −0.32)	3.67 (0.007)	0.076

^#^ Multivariable models controlling for age, sex, and presence of comorbid conditions. * The *p*-values correspond to the F-test assessing the overall model significance. Factors that were statistically significant in both the non-adjusted and adjusted models included depressive symptomatology, suicidal ideation, anxiety, and poor sleep. LWMAT: Locke and Wallace Marital Adjustment Test; CI: confidence interval; SF-12 PCS: Short-Form Health Survey Physical Component Score; SF-12 MCS: Short-Form Health Survey Mental Component Score; BDI-II: Beck’s Depression Inventory, second edition; BAI: Beck Anxiety Inventory; ISI: Insomnia Severity Index.

## Data Availability

The data that support the findings of this study are available upon request from the corresponding authors. The data are not publicly available due to privacy and ethical restrictions.
